# Extended-spectrum ß-lactamase *Providencia stuartii *in a general ICU

**DOI:** 10.1186/cc12017

**Published:** 2013-03-19

**Authors:** P Myrianthefs, E Evodia, G Fildissis, G Baltopoulos

**Affiliations:** 1 Athens University, Athens, Greece; 2 Iatriko Kentro Athinon, Athens, Greece

## Introduction

Providencia stuartii, a member of the family of Entero-bacteriacea, is a Gram-negative pathogen causing colonization and opportunistic infections in ICU patients.

## Methods

We retrospectively recorded the characteristics of P. stuartii infections in our ICU in a total period of 1 year (six-bed general ICU).

## Results

A total of 116 patients (80 males, 68.9%) were hospitalized in our ICU of mean age 58.5 ± 1.8, mean ICU stay: 23.2 ± 3.3 days, APACHE II: 19.3 ± 0.7, SAPS II: 45.7 ± 1.6, SOFA: 7.9 ± 0.4 and mortality: 18.9%. Admission diagnosis was multiple trauma (29.3%), emergency surgery (37.1%), and medical (33.6%). Of them 21 (18.1%) developed P. stuartii infection that was related to multiple trauma (P = 0.0289), length of ICU stay 69.8 ± 12.6 (median 51.0) versus 12.6 ± 1.7 (median 6.0) (P <0.0001) and illness severity APACHE II (21.7 ± 1.3 vs. 17.5 ± 0.8; P = 0.0056), SAPS II (54.9 ± 2.9 vs. 43.6 ± 1.8; P = 0.0296) and SOFA (10.6 ± 0.5 vs. 7.2 ± 0.4; P <0.0001). There was no statistically significant difference regarding sex, age or mortality (P = 0.3789). Mean day of first isolation was 31.1 ± 2.1 (median 28.0). The number of isolations per site were - blood: 11 (median day: 25.0), tracheal aspirates: 9 (median: 28.5), catheter tip: 15 (median: 31.0), urine: 6 (median: 32.0), wound: 12 (median: 25.0), biological fluids: 4 (median: 29.5), other catheters: 1 (median: 28.0), middle ear: 2 (median: 32.5), and nose: 1 (median: 25.0). Six patients had only one site isolation and the remaining 15 had multiple sites of P. stuartii isolation. We totally recorded 63 isolates of which 82.5% were second-generation and third-generation cephalosporin-resistant, 80.3% aztreonam-resistant and 81% carbapenem-resistant strains expressing an extended-spectrum ß-lactamase phenotype. All patients had previously received colistin or meropenem or tygecycline for a median period of 18.5, 15 and 10.5 days, respectively.

## Conclusion

P. stuartii infection/colonization may develop in critically ill patients, especially those with multiple trauma and prolonged ICU stay, and maybe isolated in blood after the 25th day of ICU stay. Also, infections due to extended-spectrum ß-lactamase-producing multidrug-resistant P. stuartii are an emerging problem.

